
JOURNAL INFORMATION
==============================
NLM Title Abbreviation: ScientificWorldJournal
Journal ID: ScientificWorldJournal
NLM Journal ID: 101131163
Journal ID: TSWJ

The Scientific World Journal

ISSN: 2356-6140
EISSN: 1537-744X
Publisher: Wiley

ARTICLE INFORMATION
==============================
PMCID: PMC5901284
PMID: 18040545
DOI: 10.1100/tsw.2007.250
Article ID: 102724
Article version: 1
Subjects: Case Study

Fournier's Gangrene - Findings on Computed Tomography

Cullen I. M. 1 *
Larkin J. O. 1
Moore M. 2
Fitzgerald E. 2
M. Ó'Ríordáin M. 3
Rogers E. 1
1 Department of Urology, Mercy University Hospital, Cork, Ireland
2 Department of Radiology, Mercy University Hospital, Cork, Ireland
3 Department of Surgery, Mercy University Hospital, Cork, Ireland
*I. M. Cullen: imcullen@yahoo.comAcademic Editor: Tom F. Lue

Publication date: 2007
Electronic publication date: 2007 Nov 12
Volume: 7
First page: 1839
Last page: 1841
Received 2007 May 8; Revised 2007 Aug 23; Accepted 2007 Aug 28
Copyright: Copyright © 2007 I. M. Cullen et al.
Copyright year: 2007
License: This is an open access article distributed under the Creative Commons Attribution License, which permits unrestricted use, distribution, and reproduction in any medium, provided the original work is properly cited.
License URL: https://creativecommons.org/licenses/by/3.0/

==============================
Case Study
*TheScientificWorldJOURNAL* (2007) 7, 1839–1841
TSW Urology
ISSN 1537-744X; DOI 10.1100/tsw.2007.250

TheScientificWorldJOURNAL
www.thescientificworld.com

### Fournier's Gangrene – Findings on Computed Tomography

I.M. Cullen[1*], J.O. Larkin[1], M. Moore[2], E. Fitzgerald[2], M. Ó'Ríordáin[3], and E. Rogers[1]

*[1]Department of Urology, [2]Department of Radiology, and [3]Department of Surgery, Mercy University Hospital, Cork, Ireland*

**E-mail:** imcullen@yahoo.com

*Received May 8, 2007; Revised August 23, 2007; Accepted August 28, 2007; Published November 12, 2007*

### CASE REPORT

A 47-year-old male was admitted with a 7-day history of bilateral testicular pain on a background of anorexia, nausea and vomiting, diarrhoea, and rigors. One day prior to admission, he noticed swelling and red discoloration of his penis and scrotum. There was no history of trauma, abdominal pain, urinary symptoms, or foreign travel. He had no significant medical or surgical history of note.

On examination, he was normotensive, tachycardic, and pyrexial at 38.2º. Cardiovascular, respiratory, and neurological examinations were noncontributory. Abdominal examination revealed a soft nontender abdomen with no evidence of peritonism. The scrotum and penis were markedly erythematous and oedematous, and subcutaneous emphysema (crepitus) was palpable within the scrotal skin. Both were very tender to examine, but no testicular abnormality was identified. Digital rectal examination demonstrated a very tender rectum and normal benign-feeling prostate.

Blood tests revealed a neutrophilia and elevated Erythrocyte Sedimentation Rate (ESR). Serum glucose was normal. MSU (midstream urine) test demonstrated 10 WCC and 0 RCC (white, red cell count) per high-power field. Blood cultures were taken and the patient was commenced on IV (intravenous) benzylpenicillin, metronidazole, cefuroxime, and gentamicin, together with aggressive fluid resuscitation.

A CT pelvis was performed on day 1. This demonstrated marked oedema of the scrotum and penis, with gas in the left corpus spongiosum extending into the urethra (Fig. 1). The appearances were consistent with those of Fournier's gangrene. The patient was promptly taken to theatre for scrotal debridement. A suprapubic catheter was inserted for urinary diversion, thus avoiding the affected penile urethra. Examination of the rectum under anaesthesia revealed an ischiorectal abscess, which was drained and necessitated a temporary defunctioning colostomy. The suprapubic catheter was left *in situ* for 7 days postoperatively and on removal, the patient voided normally per urethra. Thereafter, the patient made a good recovery and was duly discharged.

### DISCUSSION

Fournier's gangrene is defined as a synergistic, polymicrobial, necrotising fasciitis of the perineal, perirectal, or genital area[1]. The bacterial synergism of aerobic and anaerobic organisms, indigenous commensals below the pelvic diaphragm, results in the production of exotoxins that lead to tissue necrosis

*Corresponding author.
©2007 with author.
Published by TheScientificWorld; www.thescientificworld.com and the synthesis of insoluble subcutaneous gases that give rise to the characteristic, though not constant[2], subcutaneous emphysema of Fournier's gangrene. The bacteria involved act synergistically,

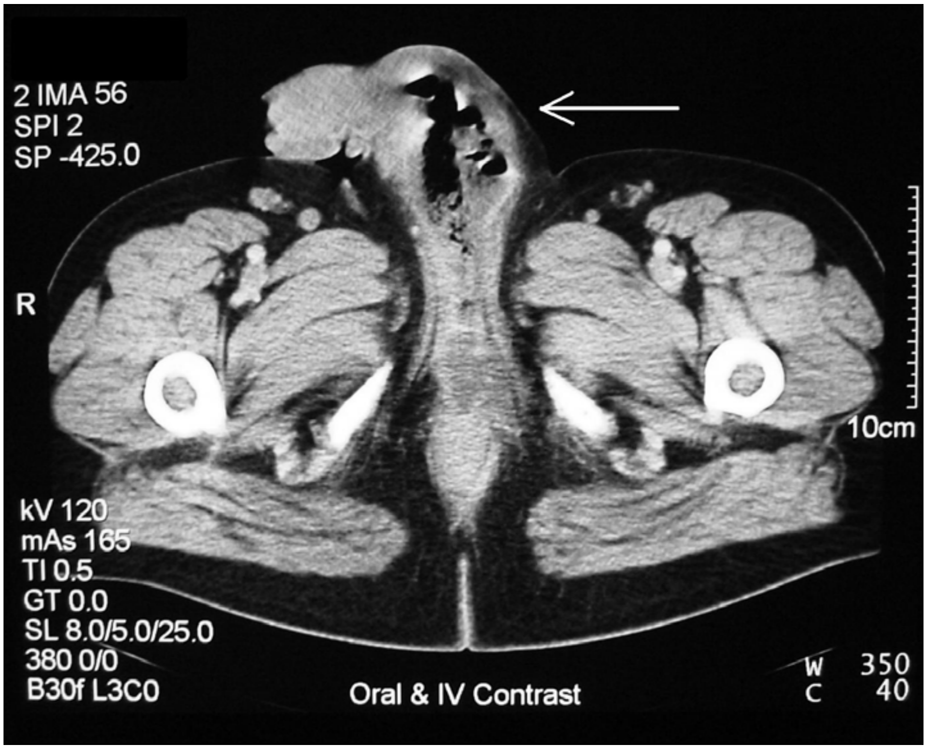

**FIGURE 1.** Demonstrating oedema of the scrotum and penis, with gas in the left corpus spongiosum extending into the urethra.

via collagenases, hyaluronidases, and other enzymes, to invade and destroy fascial planes. Ultimately, an obliterative endarteritis develops, and the ensuing cutaneous and subcutaneous vascular necrosis leads to localized ischaemia and further bacterial proliferation.

Although Fournier originally described idiopathic gangrene of the genitalia[3], Fournier's gangrene has an identifiable cause in approximately 95% of cases. The necrotizing process commonly originates with an infection in the anorectum, the urogenital tract, or the skin[4].

The onset of the condition may be insidious, however, and the initial signs, including pain, oedema, and erythema, may be difficult to distinguish clinically from cellulitis. Early diagnosis and aggressive management are essential, as overwhelming sepsis may quickly develop and is associated with a significant mortality rate – reported as 16% in one meta-analysis[4].

A deep fascia, termed Buck's fascia, covers the corpora cavernosa and the anterior urethra. Buck's fascia fuses to the dense tunica albuginea of the corpora cavernosa deep in the pelvis and this fascial layer usually limits the depth of tissue destruction in a necrotizing infection of the genitalia. The corpora cavernosa, urethra, testes, and cord structures are typically spared in Fournier's gangrene, while the superficial and deep fascia and the skin are destroyed.

Urinary diversion is accomplished with a urethral catheter in most instances. Suprapubic catheterisation is used when urethral drainage of the bladder is not possible because of pathology (e.g., stricture disease, prostatic hypertrophy). In this case report, Buck's fascia had been breached by the infective process, and the urethra and periurethral tissues were involved. Involvement of the urethra or periurethral tissues is an absolute indication for suprapubic urinary diversion to avoid further trauma to the involved delicate tissue. Immediate or delayed penectomy and orchidectomy may ultimately be necessary in cases where necrotic tissue replaces the entire penis or involves the testes at the time of surgical debridement[5].

Demonstration of soft tissue gas or detection of subcutaneous crepitance is an absolute indication for surgical exploration. One should consider imaging in suspected cases of Fournier's gangrene where clinical examination is inconclusive. The presence of gas within the soft tissues is detected more sensitively by imaging modalities when compared to physical examination.

Initial plain film may show moderate-to-large amounts of soft tissue gas. Ultrasonography (US) can be used to detect fluid or gas within the soft tissues. In addition, US can assess the blood flow to the testis if testicular torsion forms part of the differential diagnosis[1]. The drawback of US, however, is the need for direct pressure on the involved tissue; the patient with Fournier's gangrene will not tolerate this procedure.

Computed tomography (CT) imaging can detect smaller amounts of soft tissue gas than plain radiographs and can demonstrate fluid collections that track along the deep fascial planes. Distinction can be made between inflamed fascial planes or subcutaneous fat and uninvolved tissue[1]. Furthermore, CT evaluation of the perineum often demonstrates the underlying cause of Fournier's gangrene, such as perianal abscesses, fistulous tracts, incarcerated inguinal hernias, and sources of infection due to intra-abdominal and retroperitoneal processes[6,7].

The CT scan defines the extent of the disease more specifically than plain films or ultrasound, and is of greatest benefit in planning the surgical debridement central to the management of this serious condition[8,9].

### REFERENCES

1. Rajan, D.K. and Scharer, K.A. (1998) Radiology of Fournier's gangrene. *AJR Am. J. Roentgenol*. **170(1),** 163–168.
2. Baskin, L.S., Carroll, P.R., et al. (1990) Necrotising soft tissue infections of the perineum and genitalia. Bacteriology, treatment and risk assessment. *Br. J. Urol*. **65(5),** 524–529.
3. Fournier, J.A. (1883) Gangrène foudroyante de la verge. *Sem. Méd.* **2(3),** 345–347.
4. Eke, N. (2000) Fournier's gangrene: a review of 1726 cases. *Br. J. Surg*. **87(6),** 718–728.
5. Corman, J.M., Moody, J.A., et al. (1999) Fournier's gangrene in a modern surgical setting: improved survival with aggressive management. *BJU Int*. **84(1),** 85–88.
6. Ash, L. and Hale, J. (2005) CT findings of perforated rectal carcinoma presenting as Fournier's gangrene in the emergency department. *Emerg. Radiol*. **11(5),** 295–297.
7. Amendola, M.A., Casillas, J., et al. (1994) Fournier's gangrene: CT findings. *Abdom. Imaging* **19(5),** 471–474.
8. Wysoki, M.G., Santora, T.A., et al. (1997) Necrotizing fasciitis: CT characteristics. *Radiology* **203(3),** 859–863.
9. Piedra, T., Ruiz, E., et al. (2006) Fournier's gangrene: a radiologic emergency. *Abdom. Imaging* **31(4),** 500–502.

**This article should be cited as follows:**

Cullen, I.M., Larkin, J.O., Moore, M., Fitzgerald, E., Ó'Ríordáin, M., and Rogers, E. (2007) Fournier's gangrene – findings on computed tomography. *TheScientificWorldJOURNAL:* TSW Urology **7**, 1839–1841. DOI 10.1100/tsw.2007.250.
